# Clinical application of thioredoxin reductase as a novel biomarker in liver cancer

**DOI:** 10.1038/s41598-021-85688-3

**Published:** 2021-03-16

**Authors:** Xuping Wu, Qi Wang, Yousheng Lu, Jinye Zhang, Hanwei Yin, Yongxiang Yi

**Affiliations:** 1 The Second Hospital of Nanjing, Nanjing University of Chinese Medicine, Nanjing, 210000 Jiangsu China; 2 Keaise Center for Clinical Laboratory, Wuhan, China; 3 Jiangsu Provincial Cancer Hospital and Jiangsu Provincial Cancer Institute, Medical Department of Cancer Hospital Affiliated to Nanjing Medical University, Nanjing, China; 4 Nantong Tumor Hospital, Nantong, China

**Keywords:** Tumour biomarkers, Hepatocellular carcinoma, Tumour biomarkers

## Abstract

Hepatic cancer is often amenable to surgery, including percutaneous ablation, trans-arterial chemoembolization. However, in metastatic cases, surgery is often not an effective option. Chemotherapy as a conventional clinical method for treatment of malignant diseases may be useful in such cases, but it is likewise not always able to slow or halt progression, therefore novel approaches for treatment of hepatic cancer are needed. Current research suggests that molecular tumor markers (TM) can play a crucial role for diagnosis and prognostic evaluation of malignancies, and TM such as AFP, CEA, CA19-9 have been reported in many malignant diseases. Thioredoxin reductase (TrxR), a type of anti-oxidant biomarker, has become a TM of significant interest. However, little is known about the above TM and TrxR activity in liver cancer. Therefore, this paper aimed to assess these TM with regards to diagnosis and and monitoring treatment efficacy in both primary and metastatic liver cancer. Our results showed TrxR had superior performance for discriminating between liver cancer patients and healthy controls than AFP, CEA, and CA19-9. TrxR also exhibited superior performance for assessing benefits of chemotherapy regardless if patients had PLC or MLC. Meanwhile, due to diagnostic efficiency of unresponsive chemotherapy patients, TrxR also showed a higher activity levels than other general markers in liver metastasis patients. Our results suggest that application of TrxR in combination with other tumor markers may maximize the efficiency of diagnosis and assessment of therapeutic efficiency, and provide new insights for the clinical application of TrxR as a candidate biomarker for liver cancer.

## Introduction

Primary liver cancer (PLC) is a common life-threatening tumor^1,2^ with a current mortality rate close to that of lung cancer^3^. Due to the long latency period of PLC, a large number of patients reach advanced stages before diagnosis and clinical intervention^4–7^. Many patients are therefore also characterized by high recurrence and high metastasis rates^8–11^. Numerous studies have shown that several risk factors, such as excessive alcohol consumption, smoking, cirrhosis, and type 2 diabetes, predispose to PLC progression^12–15^, and these risk factors may also contribute to disease initiation. However, a main cause of PLC is viral hepatitis, which accounts for a large percentage of cases in China^16,17^. Approximately 85% of cirrhosis and liver cancer patients are diagnosed with viral hepatitis, which indicates that viral hepatitis constitutes a significant risk factor for liver cancer^18,19^.

Hepatic metastasis as an advanced stage of secondary growth carcinoma is etiologically different from PLC^20,21^. In these patients, tumors originate from distant liver metastasis through epithelial-mesenchymal transformation and circulating tumor cells, such as from esophageal and gastric cancers^22^. These tumor cells can circulate to the liver through the bloodstream, even in patients who underwent surgery^23,24^, and a large number of patients experience recurrence. Application of transcatheder arterial chemoembolization (TACE) might alleviate the progression of liver metastases to some extent^25,26^, but the treatment of invasive secondary cancers is often ineffective. Therefore, it would be helpful to improve the early detection of (metastatic) liver cancers and to evaluate the efficacy of chemotherapy with regards to survival of patients.

Timely control of tumor progression is beneficial to improving the quality of life of patients. Tumor markers have been used for almost 160 years and constitute an important clinical auxiliary tool. Specific antigens, including carbohydrate antigen (CEA) and carbohydrate antigen 19-9 (CA19-9), exhibit a partial diagnostic accuracy for some cancers^27–29^, and alpha-fetoprotein (AFP) levels are abnormally elevated in liver disease^30^. Unfortunately, the aforementioned tumor markers lack significant diagnostic value after chemotherapy, which make it difficult to evaluate disease progression (PD)^29^. Novel circulating biomarkers are therefore being explored.

TrxR is an enzyme of the triphosphopyridine nucleotide (NADPH) oxidative pathway and plays a key role in several physiological activities, such as redox pathways and DNA synthesis^31,32^. Previous studies suggested that TrxR indicates higher levels of abnormally proliferating cells, and may have a superior diagnostic efficacy than other conventional tumor markers. In addition, TrxR activity is rapidly downregulated after chemotherapy in non-small cell lung cancer (NSCLC), gastric cancer, and breast cancer^33–35^. Previous studies consistently demonstrated that TrxR has potentially high value for clinical diagnosis and assessment of therapeutic efficacy. Here, we assess the role of TrxR and other TM in primary and metastatic liver cancer (PLC and MLC, respectively). To the best of our knowledge, it is the first study to investigate the role of TrxR in the evaluation of therapeutic efficiency in the primary liver cancer (PLCs). Furthermore, this study included the comparison of TrxR activity between PLCs and MLCs, providing a new insight to the clinical appliance of TrxR in the diagnosis and monitoring therapeutic efficiency in liver cancer.

## Methods

### Patients

Cancer patients were eligible for enrollment based on histologically confirmed liver cancer, as described in Supplementary materials (Supplemental Table S1), and were consecutively recruited from The Second Hospital of Nanjing (Nanjing, China), Jiangsu Cancer Hospital (Jiangsu, China), and Nantong Tumor Hospital (Jiangsu, China). Enrollment occurred from 2017 to 2020. Sex- and age-matched health controls and patients diagnosed into other diseases (such as hepatitis) based on hematological, histopathological and computed tomography (CT) analyses^36^, were also enrolled.

### Specimen properties

Sample collection was conducted as described in the literature^34^. Extra-cellular blood samples were obtained in tubes containing EDTA or no anticoagulant for 2 h preoperatively, after which the samples followed by centrifugation at 3500 rpm for 5 min at room temperature. The upper serum plasma was collected and stored in EP tubes at 4 °C.

### The tumor marker analysis

Levels of CEA, CA19-9, and AFP as tumor markers associated with liver cancer were obtained at the indicated times during patient visits. Following previous literature^29,34^, CEA, AFP and CA19-9 were analyzed by electrochemiluminescence-based immunoassay (ECLIA) with Cobas analyzer (Roche Diagnostics, Mannheim, Germany), following the manufacturer's instructions. Reference values for all tumor markers were selected based on the Chinese Society of Clinical Oncology, and three clinical thresholds were set at 39 U/mL for CA19-9, 7.0 ng/mL for AFP, and 3.5 ng/mL for CEA^37^.

### Assay for TrxR activity analysis

The activity of thioredoxin reductase (TrxR) in plasma was measured by UV spectrophotometry as previously described in the literature^34,38–40^. The kits used were commercial kits approved for marketing and purchased from Clairvoyance Health Technology Co., Ltd, Wuhan, China. All operations were performed according to the manufacturer’s instructions^34,38,39^. A single-blinded experimental protocol was designed.

### Statistical analysis

The diagnostic efficacy of the biomarker was assessed by the receiver operator curve (ROC), assessing the value of the area under the curve (AUC), and the operating characteristics of the 95% confidence interval (CI). All statistical analyzes were performed in GraphPad Prism 7 (Graphprism, USA) and SPSS19.0 (SPSS Inc, USA). The correlation between TrxR and other tumor markers was analyzed in R by regression correlation. The non-parametric Mann–Whitney U test was applied to evaluate the difference for two parallel groups. Statistical significance was considered as *P* < 0.05^41^.

### Ethics statement

This current study, including all experimental protocols, was approved by the ethics committees of The Second Hospital of Nanjing (Nanjing, China), Jiangsu Cancer Hospital (Jiangsu, China) and Nantong Tumor Hospital (Nantong, China). The methods were carried out in accordance with the approved guidelines and regulations. Informed consent was obtained from all patients.

## Results

This retrospective analysis was carried out on 1286 specimens enrolled from Jiangsu Province Cancer Hospital, Nanjing Second People's Hospital, and Nantong Cancer Hospital between 2017 and 2020, including 327 patients with primary liver cancer before (n = 183) or after (n = 144) clinical intervention, 809 patients with liver metastases (n = 161 for therapeutic evaluation), and 150 healthy controls, respectively. Besides, 510 patients with other liver diseases [hepatitis (n = 327), liver injury (n = 61), liver dysfunction (n = 26), cirrhosis (n = 85), and fatty liver (n = 11)], were also enrolled for the comparison of TrxR levels between liver cancer and other common liver diseases.

### Pre-intervention plasma TrxR activity and AFP, CEA, and CA19-9 levels in primary liver cancer patients and healthy controls

**Figure 1:**
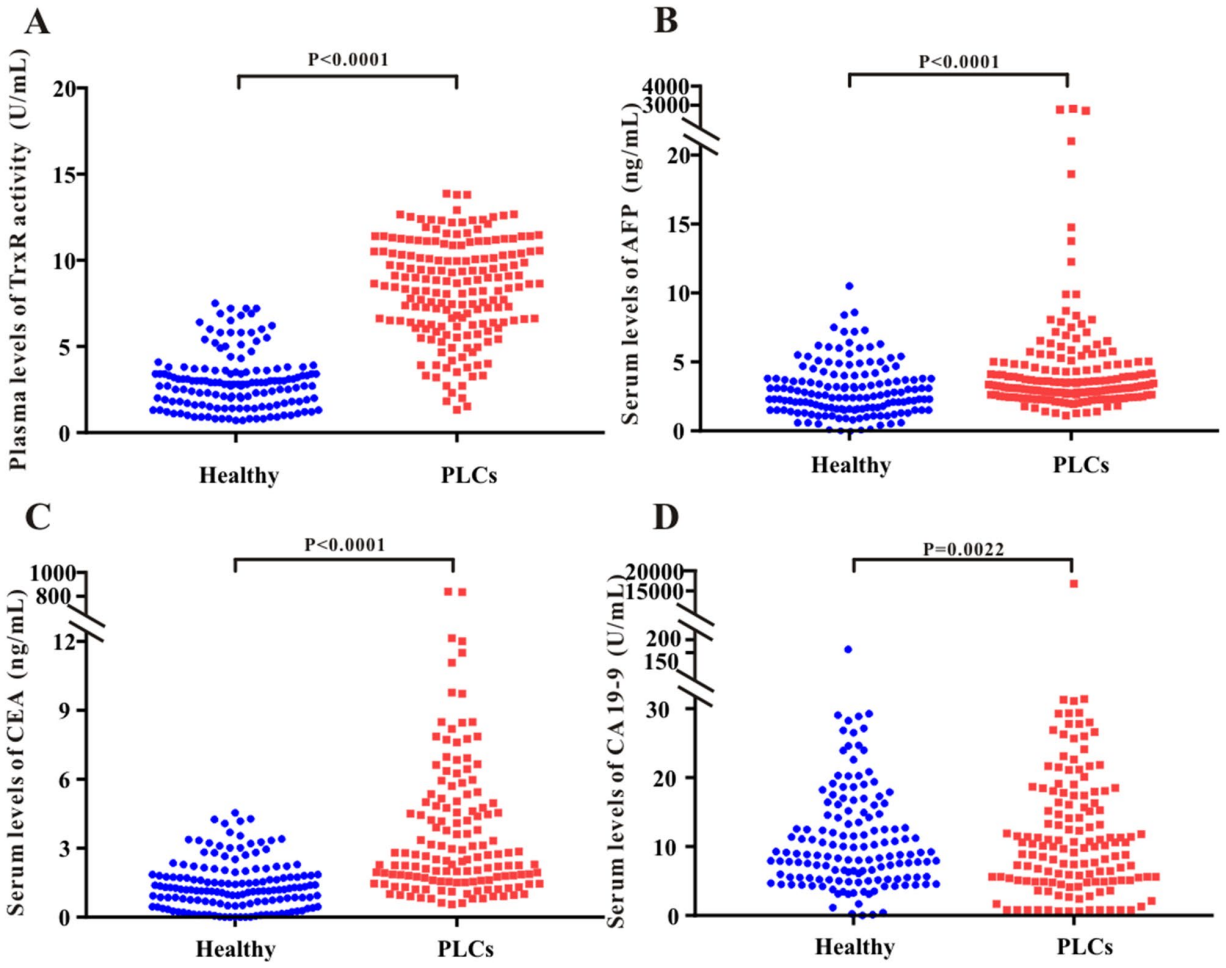
Scatter plot of the distribution of plasma TrxR (**A**), serum AFP (**B**), CEA (**C**), and CA19-9 (**D**) levels between healthy people and PLC groups before clinical interventions. P values were calculated by the nonparametric Mann–Whitney U test. Statistical significance was considered as *P* < 0.05.

To assess potential differences in TrxR activity in healthy individuals and patients with primary liver cancers (PLCs), 183 PLCs and 150 of healthy individuals were enrolled in this study. TrxR activity and levels of AFP, CEA, and CA19-9 [median (IQR)] were measured in PLCs and healthy controls before the clinical intervention. TrxR activity was significantly elevated in PLCs [8.63 (6.38, 10.05) U/mL] relative to healthy controls [2.80 (1.7, 3.6) U/mL] (Fig. 1A). Likewise, serum AFP, CEA, and CA19-9 levels were significantly increased in PLCs relative to healthy controls, indicating that TrxR activity and AFP, CEA, and CA19- are potentially sensitive biomarkers for liver cancer before clinical intervention (Fig. 1B–D).

### Potential suitability of TrxR activity as a diagnostic biomarker for primary liver cancer

**Figure 2:**
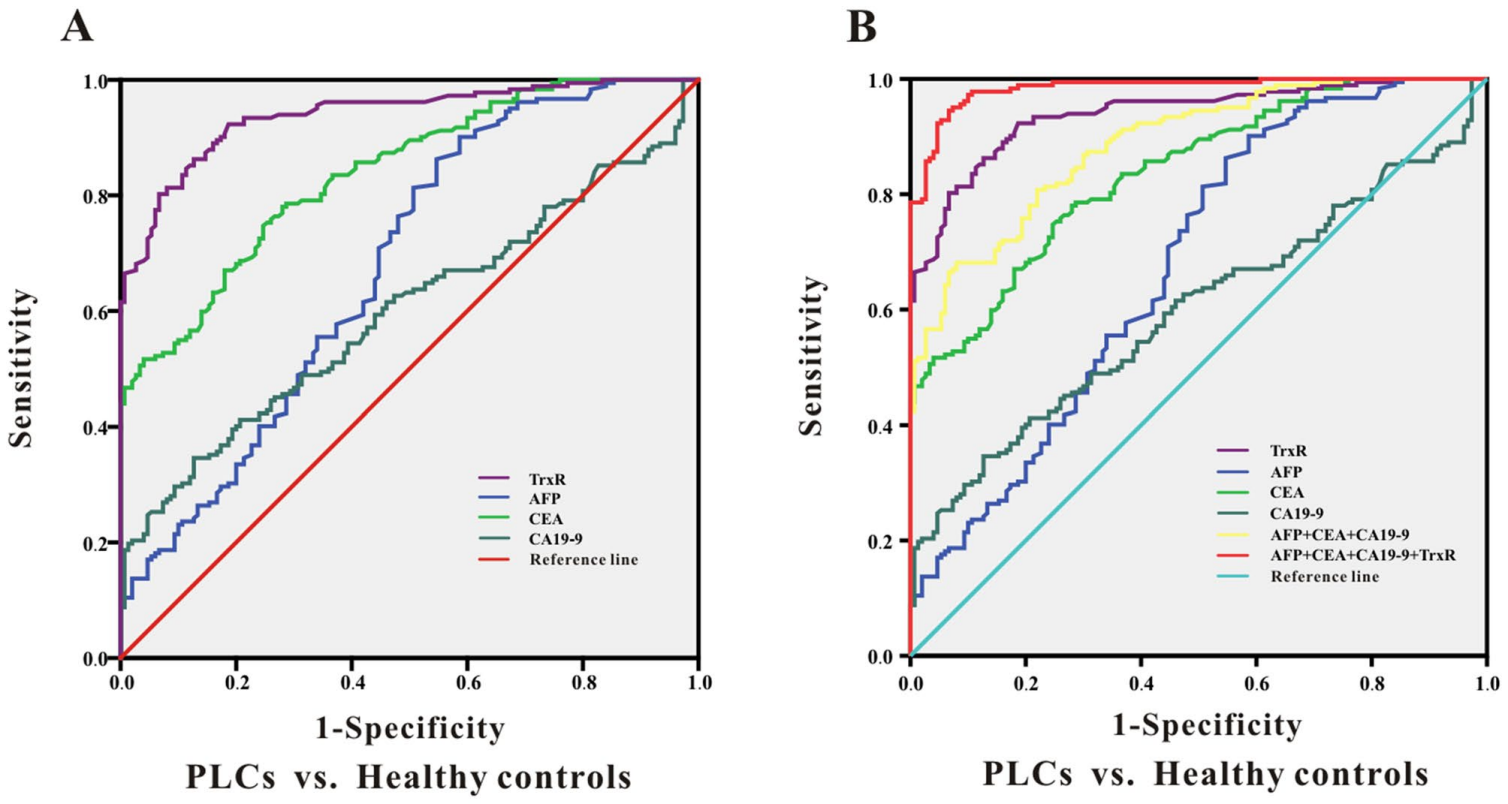
ROC curve analyses of TrxR, AFP, CEA, CA19-9 (**A**), and the combinations (**B**) for the differentiation of PLCs and healthy controls.

Analysis of ROC curves was performed to evaluate the suitability of TrxR plasma activity as a biomarker for the diagnosis of primary liver cancers (PLCs)^42^. The optimal cutoff for TrxR activity was calculated using the maximum Youden index (sensitivity + specificity-1) to distinguish PLC patients from healthy controls. As presented in Fig. 2A, the diagnotic cut-off value of TrxR activity in PLC patients was calculated to be 3.85 U/mL for a sensitivity of 92.31% and a specificity of 81.33% based on the ROC curve (AUC 0.939; 95% CI 0.915–0.964). In comparison, CEA showed the second highest AUC for distinguishing liver cancer patients from healthy controls (Table 1; 0.838; 95% CI 0.797–0.879). Simultaneously, we showed that CA19-9 and AFP have a moderate ability to discriminate between PLC patients and healthy controls, with AUCs of 0.677 (95% CI 0.618–0.735) and 0.598 (95% CI 0.537–0.658), respectively. The sensitivity of CA19-9 was less than 50%, which suggested that the use of CA19-9 and AFP for PLC diagnosis had a high risk for false negatives. It was therefore evident that TrxR activity was superior to other tumor markers for clinical diagnosis in PLC patients.

**Table 1:** The diagnostic efficiency of TrxR, CA19-9, CEA , AFP and their combinations in distinguishing PLC patients from healthy controls.

Tumor markers	PLC patients before clinical intervention						
	AUC (95%CI)	SEN%	SPE%	PPV%	NPV%	PLR	NLR
**PLC patients vs. healthy controls**							
TrxR	0.939 (0.915–0.964)	92.31	81.33	83.18	91.36	4.95	10.57
AFP	0.677 (0.618–0.735)	86.26	45.33	61.21	76.75	1.58	3.30
CEA	0.838 (0.797–0.879)	74.73	75.33	75.18	74.88	3.03	2.98
CA19-9	0.598 (0.537–0.658)	34.62	87.33	73.21	57.19	2.73	1.34
CEA + CA19-9 + AFP	0.887 (0.853–0.920)	68.13	92.00	89.49	74.27	8.52	2.89
CEA + CA19-9 + AFP + TrxR	0.984 (0.974–0.994)	94.51	93.33	93.41	94.44	14.18	16.99

SPE: specificity; SEN: sensitivity; NPV: negative predictive value; PPV: positive predictive value;NLR: negative likelihood ratio. PLR: positive likelihood ratio; the diagnosic threshold of TrxR activity was 3.85 U/mL.

Furthermore, combinations of CA19-9, CEA and AFP displayed an increased efficacy for detecting PLC patients (AUC 0.887; 95% CI 0.853–0.920) compared with the three individual levels (*P* < 0.5). Remarkably, by adding TrxR to this combination group, there was further improvement in the diagnostic efficiency for PLC (AUC 0.984; 95% CI 0.887–0.984). These results provide a promising diagnostic combination of four biomarkers for PLC diagnosis, which could be used for future clinical applications (Fig. 2B and Table 1).

Additionally, we also investigated the level of TrxR activity in other liver diseases including hepatitis, liver injury, liver dysfunction, cirrhosis, and fatty liver. As shown in Supplemental Fig. S1, other liver diseases also showed lower levels of TrxR activity compared to PLCs, suggesting TrxR level was specifically elevated in PLCs instead of other liver diseases.

### Assessment of therapeutic efficacy by monitoring TrxR activity after chemotherapy in PLC patients

To further investigate TrxR activity with regards to response to chemotherapy in patients with PLC, two groups of 144 PLC patients were divided based on clinical results. These patients were classified as Clinical Unresponsive Patients (CUP, 49 patients) or Clinical Responsive Patients (CRP, 95 patients) according to CT results. Patients with complete response (CR), partial response (PR) or stable disease (SD) mostly benefted from the chemotherapy and were included into CRP group. On the contrary, patients with progressive disease (PD) or uncontrolled condition afer chemotherapy were included into CUP group.Further statistical analysis was performed by measuring plasma TrxR levels in the CUP and CRP groups.

Firstly, we investigated if TrxR activity is an independent indicator for the diagnosis and therapeutic evaluation in liver cancer. As shown in Supplemental Fig. S2, correlation analysis indicated no significant correlation between TrxR activity and CEA, CA19-9, or AFP in either healthy group or liver patients. Thus, TrxR activity can be considered as an independent indicator for the diagnosis and therapeutic evaluation in liver cancer, and TrxR level was not affected by other TMs. Meanwhile, a detailed analysis has been performed to compare the TrxR activity among different histological types of PLCs. As shown in Supplemental Fig. S3, TrxR activity was not significantly different among hepatocellular carcinoma (HCCs), intrahepatic cholangiocarcinoma (ICCs), and combined HCCs/ICCs in PLCs either before clinical interventions or after chemotherapy, suggesting that TrxR levels in PLCs were not affected by the histological types of primary liver cancer.

**Figure 3:**
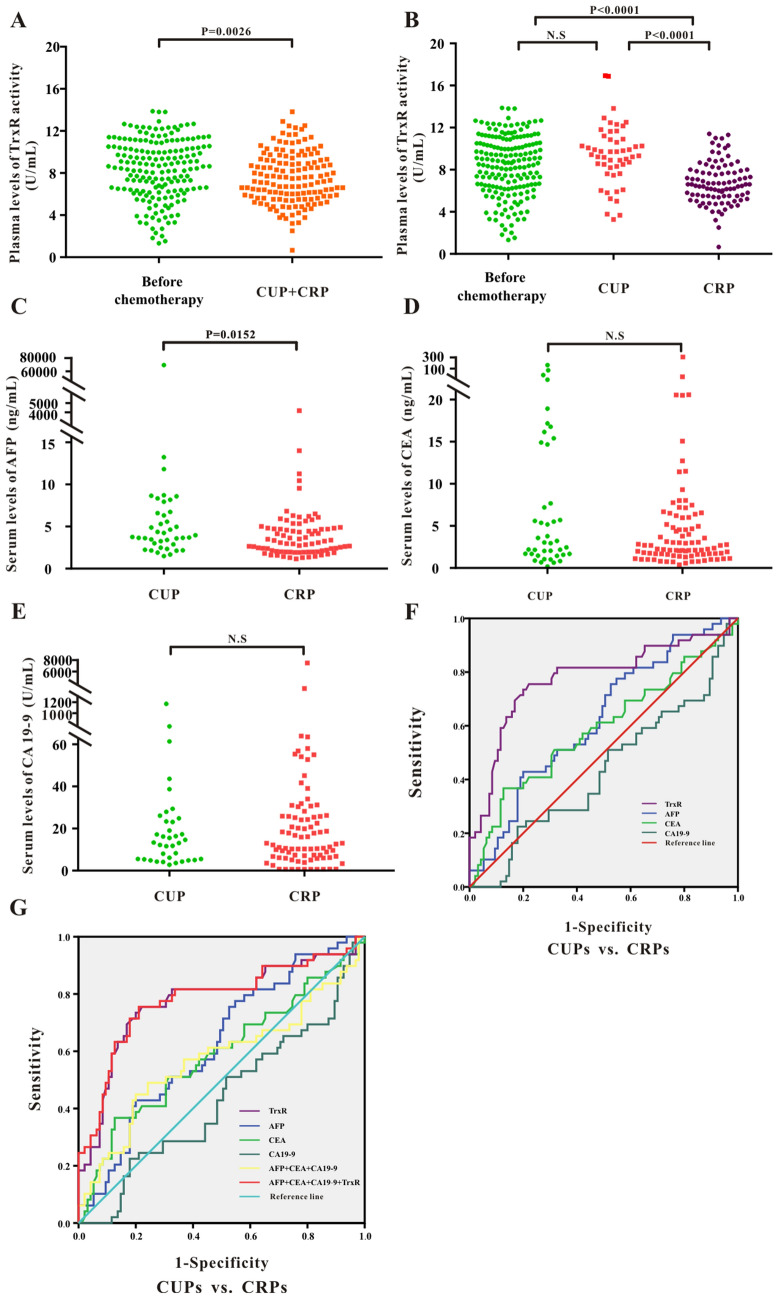
(**A**) Scatter plot of the distribution of plasma TrxR activity levels between liver cancer patients before clinical interventions and PLC patients after chemotherapy. (**B**–**E**) Scatter plot of the distribution of plasma TrxR (**B**), serum AFP (**C**), CEA (**D**), and CA19-9 (**E**) among PLC patients with different clinical outcome after chemotherapy (CUP vs. CRP). CUP: clinical unresponsive patient; CRP: clinical responsive patients. P values were determined by the Mann–Whitney U test. N.S: no statistical significance. (**F**–**G**) ROC curve analyses of TrxR, AFP, CEA, CA19-9 (**F**), and the combinations (**G**) for the differentiation of PLCs with different clinical outcome after chemotherapy (CUP vs. CRP).

Figure 3A shows overall post-chemotherapy TrxR levels in the PLC group [7.09 (5.90, 8.87) U/mL], which were lower than in pre-intervention patients [8.63 (6.38, 10.50) U/mL]. Notably, TrxR activity levels in the CRP group [6.60 (5.70, 8.20) U/mL] were significantly decreased compared with the CUP group [8.80 (7.53, 10.23) U/mL], indicating that TrxR levels decrease in PLC patients benefitting of chemotherapy (Fig. 3B). The TrxR levels in the CUP group remained unchanged compared with patients before the clinical intervention. Consistent with TrxR activity, AFP levels were also reduced in CRP patients compared with CUP patients (Fig. 3C). Downregulation of other tumor markers, including CEA and CA19-9 levels was not significantly different between the CRP and CUP groups (Fig. 3D,E and Supplemental Fig. S4).

To further confirm that TrxR offers effective therapeutic evaluation in PLC patients after chemotherapy, we analysed the therapeutic value by ROC curve. As shown in Fig. 3F and Table 2, the threshold for TrxR activity to discriminate between CUPs and CRPs was calculated at 7.45 U/mL, with a sensitivity of 73.74% and a specificity of 80.00% (AUC 0.776; 95% CI 0.687–0.865). Meanwhile, AFP showed the second highest AUC level (AUC 0.624; 95% CI 0.529–0.718) in distinguishing CUPs from CRPs. CA19-9 and CEA had very limited ability to discriminate between CUPs and CRPs, with corresponding AUCs of 0.433 (95% CI 0.333–0.534) and 0.588 (95% CI 0.485–0.691). The sensitivity of CA19-9 and CEA was less than 50%, which indicates that CA19-9 and CEA for therapeutic evaluation had a high risk for false negatives. When adding TrxR to a combination panel of AFP, CA19-9 and CEA, the value of therapeutic evaluation in patients with PLC was further strengthened relative to TrxR alone or to the other three biomarker combination panel (AUC 0.781; 95% CI 0.693–0.869) (Fig. 3G and Table 2). In summary, the ability to monitor therapeutic evaluation by plasma TrxR activity was superior to CEA, CA19-9 and AFP, but combination of all four markers exhibited the highest AUC.

**Table 2:** The assessment of therapeutic efficiency by TrxR, AFP, CA19-9, and CEA and their combinations after chemotherapy in PLC patients.

Tumor markers	PLCs after chemotherapy						
	AUC (95%CI)	SEN%	SPE%	PPV%	NPV%	PLR	NLR
**Primary carcinoma of liver: CUPs vs.CRPs**							
TrxR	0.776 (0.687–0.865)	73.47	80.00	78.60	75.10	3.67	3.02
AFP	0.624 (0.529–0.718)	75.51	47.37	58.93	65.92	1.43	1.93
CEA	0.588 (0.485–0.691)	36.73	87.37	74.41	58.00	2.91	1.38
CA19-9	0.433 (0.333–0.534)	22.45	82.11	55.64	51.43	1.25	1.06
CEA + CA19-9 + AFP	0.574 (0.466–0.681)	44.90	80.00	69.18	59.21	2.24	1.45
CEA + CA19-9 + AFP + TrxR	0.781 (0.693–0.869)	75.51	78.95	78.20	76.32	3.59	3.22

SPE: specificity; SEN: sensitivity; NPV: negative predictive value; PPV: positive predictive value;NLR: negative likelihood ratio. PLR: positive likelihood ratio; the diagnosic threshold of TrxR activity level was 7.45 U/mL in PLC patients.

### Assessment of therapeutic efficacy by monitoring TrxR activity after chemotherapy in MLC patients

**Figure 4:**
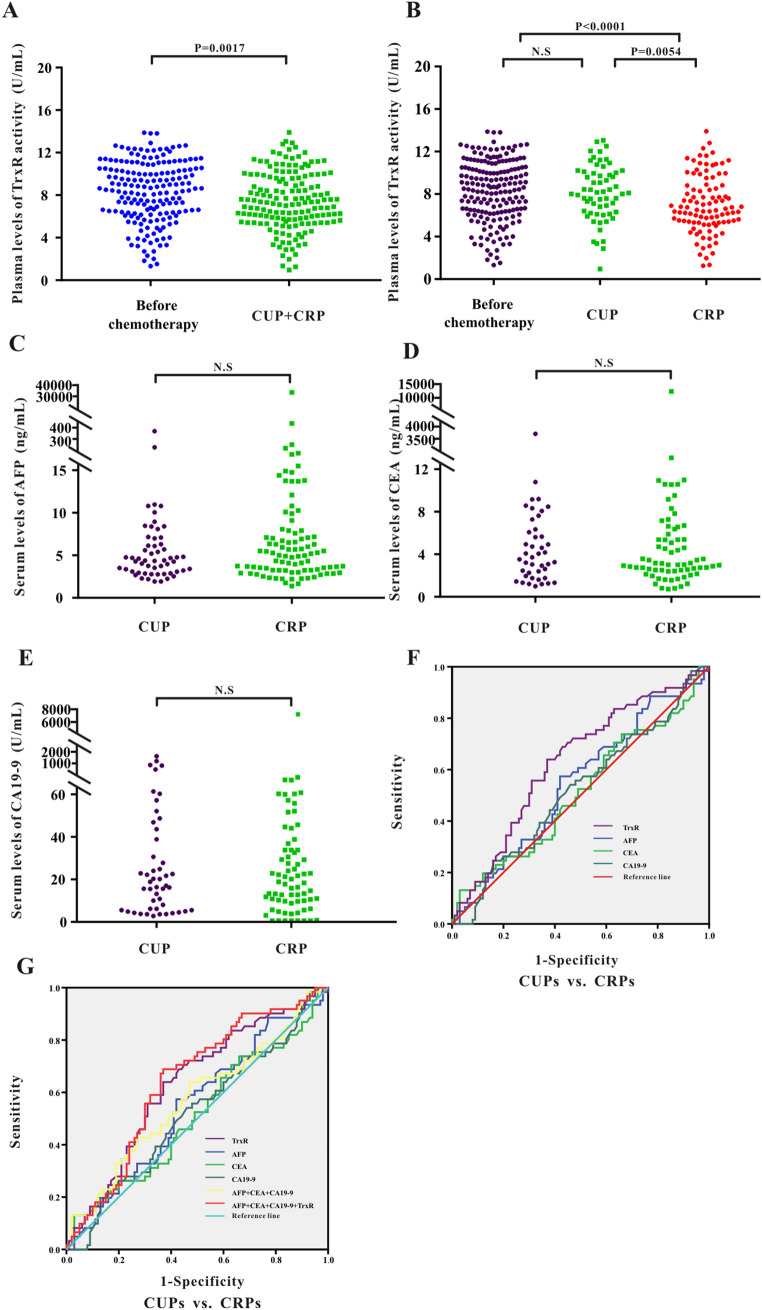
(**A**) Scatter plot of the distribution of plasma TrxR activity levels between liver cancer patients before clinical interventions and MLC patients after chemotherapy. (**B-E**) Scatter plot of the distribution of plasma TrxR (**B**), serum AFP (**C**), CEA (**D**), and CA19-9 (**E**) among MLC patients with different clinical outcome after chemotherapy (CUP vs. CRP). CUP: clinical unresponsive patient; CRP: clinical responsive patients. P values were determined by the Mann–Whitney U test. N.S: no statistical significance. (**F-G**) ROC curve analyses of TrxR, AFP, CEA, CA19-9 (**F**), and the combinations (**G**) for the differentiation of MLCs with different clinical outcome after chemotherapy (CUP vs. CRP).

Above studies have investigated the relevance between TrxR and PLCs, however, so far it was not known whether TrxR is associated with the prognosis of metastatic liver cancer (MLCs). As shown in Supplemental Fig. S5, TrxR activity was not significantly different among MLCs originated from different tumor entities, which includes intestinal cancer, gastric cancer, breast cancer, lung cancer, pancreatic cancer, esophageal cancer and nasopharyngeal cancer, suggesting that TrxR levels in MLCs were not affected by the primary organs of the tumor. Consistent with the observation in PLCs, analysis of TrxR activity revealed a significant decrease after chemotherapy [7.35(5.62, 9.79) U/mL] in MLCs compared with those before clinical intervention [8.63 (6.38, 10.50) U/mL] (Fig. 4A).

Image-based methods such as CT were often used to observe the efficacy of chemotherapy for metastatic liver cancer^42,43^. Here in MLC patients, the decrease in TrxR activity was greater in the CRP group [7.70 (5.78, 10.21) U/mL] than in the CUP group [9.40 (7.55, 11.27) U/mL] (Fig. 4B). However, other TMs such as CEA, CA19-9 and AFP, were not significantly different between CRP and CUP groups in MLCs (Fig. 4C–E and Supplemental Fig. S6), suggesting TrxR exerted a significant advantage over other TMs in the therapeutic evaluation of chemotherapy in MLCs. Similar to the observations in PLC patients, plasma TrxR activity exhibited higher sensitivity and specificity (AUC 0.630; 95% CI 0.542–0.718) than CEA, CA19-9 and AFP levels in discrimination between CRPs and CUPs in MLCs based on ROC analysis (Fig. 4F and Table 3). Application of TrxR in combination with other TMs enhanced the sensitivity and specificity of assessment of therapeutic efficacy (AUC 0.643; 95% CI 0.556–0.729) compared with the other tumor markers alone (Fig. 4G and Table 3). The above results provide an insight for evaluating the efficiency of chemotherapy in MLC patients using biomarkers. TrxR appears to play an important role as a novel serum biomarker and was able to effectively assess therapeutic efficacy in hepatic metastasis patients.

**Table 3:** Assessment of therapeutic efficiency using TrxR, AFP, CA19-9, CEA and their combinations after chemotherapy in MLC patients.

Tumor markers	MLCs after chemotherapy						
	AUC (95%CI)	SEN%	SPE%	PPV%	NPV%	PLR	NLR
**Metastatic carcinoma of liver: CUPs vs. CRPs**							
TrxR	0.630 (0.542–0.718)	63.93	63.00	63.34	63.59	1.73	1.75
AFP	0.544 (0.453–0.636)	57.38	58.00	57.74	57.64	1.37	1.36
CEA	0.511 (0.417–0.605)	13.11	97.00	81.38	52.75	4.37	1.12
CA19-9	0.519 (0.427–0.611)	24.59	84.00	60.58	52.69	1.54	1.11
CEA + CA19-9 + AFP	0.570 (0.476–0.663)	63.93	53.00	57.63	59.51	1.36	1.47
CEA + CA19-9 + AFP + TrxR	0.643 (0.556–0.729)	68.85	63.00	65.05	66.92	1.86	2.02

SPE: specificity; SEN: sensitivity; NPV: negative predictive value; PPV: positive predictive value;NLR: negative likelihood ratio. PLR: positive likelihood ratio; the diagnosic threshold of TrxR activity level was 8.285 U/mL in MLC patients.

### TrxR, AFP, CA19-9 and CEA were elevated in MLC patients compared to PLC patients in both CUP and CRP group

Within the CUP or CRP group, it was crucial to further compare biomarkers and combination panels between the MLC and PLC groups to explore if the therapeutic efficiency of TrxR and other TMs were affected by the type of liver cancer.

**Figure 5:**
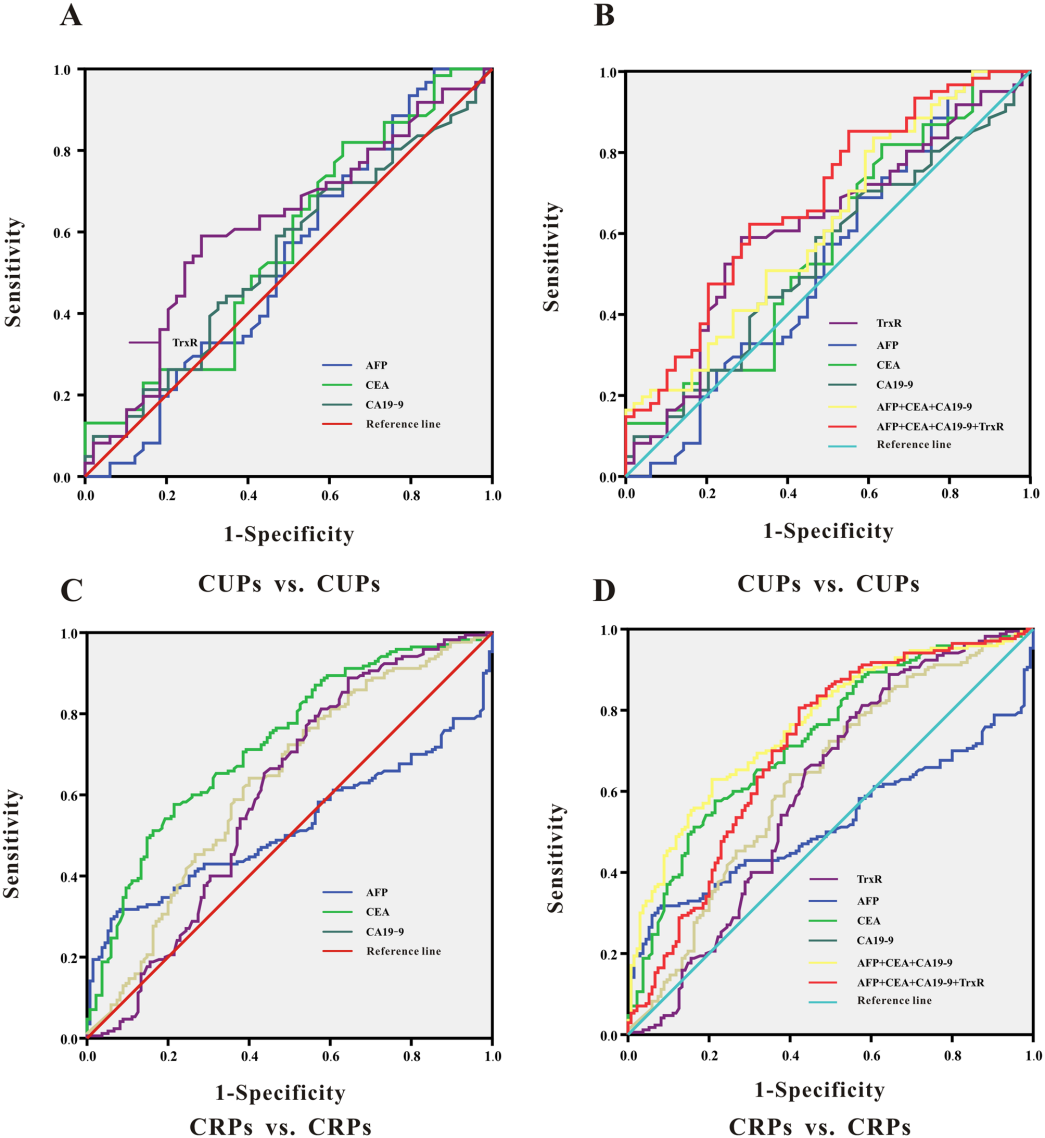
(**A**, **B**) ROC curve analyses of TrxR, AFP, CEA, CA19-9 (**A**), and the combinations (**B**) for the differentiation of CUPs with different cancer types (PLCs vs. MLCs). (**C**, **D**) ROC curve analyses of TrxR, AFP, CEA, CA19-9 (**C**), and the combinations (**D**) for the differentiation of CRPs with different cancer types (PLCs vs. MLCs).

Among all CUP patients, as shown in Fig. 5A,B and Table 4, TrxR activities and other TMs in MLC patients were significantly higher than those in PLC patients. The diagnostic cut-off value of TrxR activity to distinguish between PLC and MLC patients was calculated at 8.48 U/mL, with a sensitivity of 59.02% and a specificity of 71.43% based on ROC results (AUC 0.615; 95% CI 0.508–0.721). The combination of TrxR with other TMs could significantly improve the diagnostic sensitivity in the CUP group (AUC 0.683; 95% CI 0.583–0.783), suggesting that levels of TrxR and other TMs were significantly higher in MLCs compared with PLCs in CUP group. Consistently, in CRP group, TrxR activities in MLC patients were also remarkably higher than those in PLC patients, with a sensitivity of 88.82% and a specificity of 35.56% based on ROC results (AUC 0.605; 95% CI 0.538–0.718) (Fig. 5C,D and Table 4). Collectively, these data suggested that in both CRP and CUP groups, all these TM markers (TrxR, CEA, CA19-9, AFP) were consistently elevated in MLCs in comparison with PLCs.

**Table 4:** Assessment of therapuetic efficiency by TrxR, CEA, AFP, and CA19-9 and their combinations in distinguishing MLC from PLC patients in the CUP and CRP group.

Tumor markers	Liver cancer patients after chemotherapy						
	AUC (95%CI)	SEN%	SPE%	PPV%	NPV%	PLR	NLR
**CUPs for differences between PLCs and MLCs**							
TrxR	0.615 (0.508–0.721)	59.02	71.43	67.38	63.54	2.07	1.74
AFP	0.529 (0.417–0.642)	100.00	14.29	53.85	100.00	1.17	—
CEA	0.568 (0.459–0.678)	81.97	36.73	56.44	67.07	1.30	2.04
CA19-9	0.540 (0.431–0.648)	59.02	53.06	55.70	56.42	1.26	1.29
CEA + CA19-9 + AFP	0.618(0.513–0.724)	83.61	38.78	57.73	70.29	1.37	2.37
CEA + CA19-9 + AFP + TrxR	0.683 (0.583–0.783)	62.30	69.39	67.05	64.79	2.03	1.84
**CRPs for differences between PLCs and MLCs**							
TrxR	0.605 (0.538–0.718)	88.82	35.56	57.95	76.08	1.38	3.18
AFP	0.522 (0.456–0.587)	31.18	92.59	80.80	57.36	4.21	1.35
CEA	0.728 (0.672–0.785)	57.65	78.52	72.85	64.96	2.68	1.85
CA19-9	0.631 (0.567–0.695)	64.12	60.00	61.58	62.58	1.60	1.67
CEA + CA19-9 + AFP	0.763 (0.771–0.816)	62.94	79.26	75.21	68.14	3.03	2.14
CEA + CA19-9 + AFP + TrxR	0.705 (0.645–0.766)	80.59	57.78	65.62	74.85	1.91	2.98

SPE: specificity; SEN: sensitivity; NPV: negative predictive value; PPV: positive predictive value;NLR: negative likelihood ratio. PLR: positive likelihood ratio.

## Discussion

Primary liver cancer (PLC) is a common clinical malignant tumor^44,45^ with high mortality rates especially in western developed countries^16,46^. The causes of PLC are so far not completely elucidated. It is generally considered that PLC is closely associated with viral hepatitis, liver cirrhosis, excessive drinking, and other risk factors^47,48^. Most of the patients are diagnosed at low-differentiated stages, which seriously impacts the prognosis of these patients^49^. Screening tests and timely treatment could significantly improve the quality of life and survival in PLC patients. Different from the pathogenesis of PLC, patients with liver metastasis as secondary liver tumors develop these tumors as distal metastasis of other cancers, such as colorectal cancer^50,51^. In these cases, surgical resection alone may offer little benefit, and chemotherapy is the main therapeutic strategy for advanced patients^52^. However, the clinical benefits of liver metastasis chemotherapy are not always obvious and multiple chemotherapy leads to an additional burden of patients due to adverse effects^52,53^. Therefore, the therapeutic response of patients with liver metastasis and timely control of the progression of liver metastases are of great significance to improve the survival and clinical benefits of patients with liver metastasis.

Tumor markers have become an important auxiliary tool for clinical diagnosis^54^. It has been reported that CA19-9 has high accuracy and detection efficiency as a tumor marker for the diagnosis of pancreatic cancer and liver cancer^55,56^. CEA also offers diagnostic value for liver cancer, breast cancer, intestinal cancer, and other malignant tumors^27^. The expression of AFP is elevated in liver cirrhosis and liver cancer^57^. However, although these tumor markers offer certain diagnostic value for liver cancer, they offer limited value for assessing therapeutic efficacy following chemotherapy and thus new strategies are being developed.

Thioredoxin reductase (TrxR) is a NADPH-dependent dimeric selenide containing FAD domains. Numerous studies have shown that levels of TrxR are significantly higher than those of CEA and CA19-9 in malignant diseases such as breast, lung, and colorectal cancers^34,58^. In addition, TrxR has shown great therapeutic value in malignant diseases such as renal and lung cancer, and the combined sensitivity of TrxR compared with conventional tumor markers alone seems to be superior^34^. Therefore, the combination of TrxR with other conventional tumor markers has important clinical implications for the diagnosis and monitoring of therapeutic efficacy.

To further study the diagnostic value of TrxR and conventional tumor markers for liver cancer, this study assessed TrxR and CEA, CA19-9, AFP in a primary liver cancer group and a healthy control group. The results show that TR activity was significantly enhanced in PLC patients compared with healthy controls (Fig. 1A and Table 1). Other tumor markers, including CEA and AFP, also had a high diagnostic sensitivity, which could therefore also be used to distinguish the liver cancer patients and healthy controls. The diagnostic value of CA19-9 was not as high, which may be due to the fact the control group included people with chronic basic diseases, such as chronic pharyngitis and diabetes. In addition, we also used TrxR combined with conventional tumor markers to evaluate the diagnostic efficacy of liver cancers (Fig. 2B), which showed the highest performance of markers assessed in this study.

Analysis of the levels of TMs which indicate clinical response may also be of great value to monitor the therapeutic efficacy after chemotherapy. The role of TrxR levels in liver cancer following chemotherapy was so far unknown. Here, we showed that TrxR levels decreased following chemotherapy in both PLC and MLC (Figs. 3A, 4A). The levels of TrxR and AFP were lower in the CRP group compared with those in the CUP group, while other tumor markers showed no significant difference before and after chemotherapy (Figs. 3B–E, 4B–E). These results revealed a suitability of TrxR for monitoring chemotherapy response. Furthermore, plasma TrxR activity and combination panels had an obvious effect on improving assessment of therapeutic efficiency over individual TM levels (Tables 2, 3). These results suggested that AFP and other conventional tumor markers alone may not be sufficient to distinguish CRP from CUP patients. Combination of TrxR and the other TMs significantly improved diagnostic efficiency in both MLC and PLC patients. Regardless of clinical outcome (CUP or CRP), the TrxR levels were significantly elevated in MLCs compared with PLCs.

In summary, this is the first study to identify the role of TrxR activity in the diagnosis and therapeutic evaluation of liver cancer. According to our results, TrxR activity was generally more effective than other routine tumor markers including AFP, CEA, and CA19-9 in the diagnosis and monitoring the therapeutic efficiencies in both PLCs and MLCs. Combination of TrxR with other TMs may significantly enhance the clinical diagnostic value and assessment of therapeutic efficacy of liver cancer patients. Furthermore, this study included the comparison of TrxR activity between PLCs and MLCs, providing a new insight to the clinical appliance of TrxR in the diagnosis and monitoring therapeutic efficiency in liver cancer. Among common liver diseases, TrxR level was specifically elevated in primary liver cancer compared with other liver diseases or healthy controls, suggesting the upregulation of TrxR is only sensitive to the carcinogenesis in liver, and can be considered as a promising auxiliary tool in the clinical diagnosis of liver cancer. Taken together, this study has revealed TrxR as a novel biomarker in liver cancer with strong clinical relevance, suggesting TrxR as a promising tool in future clinical application of liver cancer diagnosis and therapeutic evaluation.

## Supplementary Information

Supplementary Information

## References

[1] Li C, Li G, Miao R, Lu X, Zhong S, Sang X (2012). Primary liver cancer presenting as pyogenic liver abscess: Characteristics, diagnosis, and management. J. Surg. Oncol..

[2] Frangov T, Tasev V, Bulanov D, Gaĭdarski R, Dimitrova V (2003). Postoperative liver failure after hepatic resections for hepatocellular carcinoma. Khirurgiia.

[3] Bray F, Ferlay J, Soerjomataram I, Siegel RL, Torre LA, Jemal A (2018). Global cancer statistics 2018: GLOBOCAN estimates of incidence and mortality worldwide for 36 cancers in 185 countries. CA.

[4] De Castro J, Gonzalez-Larriba JL, Vazquez S, Massuti B, Sanchez-Torres JM, Domine M (2017). Long-term survival in advanced non-squamous NSCLC patients treated with first-line bevacizumab-based therapy. Clin. Transl. Oncol..

[5] Arends J, Bachmann P, Baracos V, Barthelemy N, Bertz H, Bozzetti F (2017). ESPEN guidelines on nutrition in cancer patients. Clin. Nutr..

[6] Zimmermann C, Swami N, Krzyzanowska M, Hannon B, Leighl N, Oza A (2014). Early palliative care for patients with advanced cancer: a cluster-randomised controlled trial. Lancet.

[7] El Jabbour T, Lagana S, Lee H (2019). Update on hepatocellular carcinoma: Pathologists' review. World J. Gastroentero..

[8] Lee YJ, Park H, Kang CM, Gwark S-C, Lee SB, Kim J (2019). Risk stratification system for groups with a low, intermediate. Cancer Res. Treat..

[9] Serrano PE, Gu C-S, Husien M, Jalink D, Ritter A, Martel G (2019). Risk factors for survival following recurrence after first liver resection for colorectal cancer liver metastases. J. Surg. Oncol..

[10] Wakizaka K, Yokoo H, Kamiyama T, Ohira M, Kato K, Fujii Y (2019). Clinical and pathological features of combined hepatocellular–cholangiocarcinoma compared with other liver cancers. J. Gastroen Hepatol..

[11] Llovet JM, Zucman-Rossi J, Pikarsky E, Sangro B, Schwartz M, Sherman M (2016). Hepatocellular carcinoma. Nat. Rev. Dis. Primers.

[12] Mehta G, Macdonald S, Cronberg A, Rosselli M, Khera-Butler T, Sumpter C (2018). Short-term abstinence from alcohol and changes in cardiovascular risk factors, liver function tests and cancer-related growth factors: a prospective observational study. BMJ Open.

[13] Yu H, Harris RE, Kabat GC, Wynder EL (1988). Cigarette smoking, alcohol consumption and primary liver cancer: A case-control study in the USA. Int. J. Cancer.

[14] Zhang X-X, Wang L-F, Jin L (2015). Primary biliary cirrhosis-associated hepatocellular carcinoma in Chinese patients: incidence and risk factors. World J. Gastroentero..

[15] Su Q, Sun F, Li J, Zhang H, Qiao L (2015). The correlation analysis of primary liver cancer with Type 2 diabetes. Indian J. Cancer.

[16] Collaboration GBoDLC. The Burden of Primary Liver Cancer and Underlying Etiologies From 1990 to 2015 at the Global, Regional, and National Level: Results From the Global Burden of Disease Study 2015. *JAMA Oncol.* 3, 1683–1691. https://doi.org/10.1001/jamaoncol.2017.3055 (2017) 10.1001/jamaoncol.2017.3055 PMC5824275 28983565

[17] Cui YA, Moriyama M, Chayama K, Liu Y, Ya C, Muzembo BA (2019). Efficacy of a self-management program in patients with chronic viral hepatitis in China. BMC Nurs..

[18] van Gemert C, Wang J, Simmons J, Cowie B, Boyle D, Stoove M (2016). Improving the identification of priority populations to increase hepatitis B testing rates, 2012. BMC Public Health.

[19] Batyrbekova N, Aleman S, Lybeck C, Montgomery S, Duberg A-S (2020). Hepatitis C virus infection and the temporal trends in the risk of liver cancer: a national register-based cohort study in Sweden. Cancer Epidem. Biomar..

[20] Zhou XD (2002). Recurrence and metastasis of hepatocellular carcinoma: progress and prospects. Hepatob. Pancreat Dis..

[21] Liu Q, Zhang A, Xu W, Dong J (2011). A new view of the roles of blood flow dynamics and Kupffer cell in intra-hepatic metastasis of hepatocellular carcinoma. Med. Hypotheses..

[22] Hayes T, Smyth E, Riddell A, Allum W (2017). Staging in esophageal and gastric cancers. Hematol. Oncol. Clin. North Am..

[23] Kamel SI, de Jong MC, Schulick RD, Diaz-Montes TP, Wolfgang CL, Hirose K (2011). The role of liver-directed surgery in patients with hepatic metastasis from a gynecologic primary carcinoma. World J. Surg..

[24] Shin H, Kim CW, Lee JL, Yoon YS, Park IJ, Lim S-B (2019). Solitary colorectal liver metastasis after curative intent surgery: prognostic factors affecting outcomes and survival. ANZ J. Surg..

[25] Kodama K, Kawaoka T, Aikata H, Uchikawa S, Inagaki Y, Hatooka M (2018). Comparison of clinical outcome of hepatic arterial infusion chemotherapy and sorafenib for advanced hepatocellular carcinoma according to macrovascular invasion and transcatheter arterial chemoembolization refractory status. J. Gastroen. Astroen. Hepatol..

[26] Hiraoka A, Kumada T, Kudo M, Hirooka M, Koizumi Y, Hiasa Y (2017). Hepatic function during repeated TACE procedures and prognosis after introducing sorafenib in patients with unresectable hepatocellular carcinoma: Multicenter analysis. Digest Dis..

[27] Hao C, Zhang G, Zhang L (2019). Serum CEA levels in 49 different types of cancer and noncancer diseases. Prog. Mol. Biol. Transl..

[28] Wang W, Xu X, Tian B (2017). The diagnostic value of serum tumor markers CEA, CA19-9, CA125, CA15-3, and TPS in metastatic breast cancer. Clin. Chim. Acta.

[29] Feng F, Tian Y, Xu G, Liu Z, Liu S, Zheng G (2017). Diagnostic and prognostic value of CEA, CA19-9, AFP and CA125 for early gastric cancer. BMC Cancer.

[30] Galle PR, Foerster F, Kudo M, Chan SL, Llovet JM, Qin S (2019). Biology and significance of alpha-fetoprotein in hepatocellular carcinoma. Liver Int..

[31] Arner ES, Holmgren A (2006). The thioredoxin system in cancer. Semin. Cancer Biol..

[32] Ouyang Y, Peng Y, Li J (2018). Modulation of thiol-dependent redox system by metal ions via thioredoxin and glutaredoxin systems. Metallomics.

[33] Peng W, Zhou Z, Zhong Y, Sun Y, Wang Y, Zhu Z (2019). Plasma activity of thioredoxin reductase as a novel biomarker in gastric cancer. Sci. Rep..

[34] Ye S, Chen X, Yao Y, Li Y, Sun R, Zeng H (2019). Thioredoxin reductase as a novel and efficient plasma biomarker for the detection of non-small cell lung cancer: a large-scale, multicenter study. Sci. Rep..

[35] Bhatia M, McGrath KL, Di Trapani G, Charoentong P, Shah F, King MM (2016). The thioredoxin system in breast cancer cell invasion and migration. Redox Biol..

[36] Flejou JF (2011). WHO: Classification of digestive tumors: the fourth edition. Ann. Pathol..

[37] Cong WM, Bu H, Chen J, Dong H, Committee G (2016). Practice guidelines for the pathological diagnosis of primary liver cancer: 2015 update. World J. Gastroentero..

[38] Chen G, Chen Q, Zeng F, Zeng L, Yang H, Xiong Y (2017). The serum activity of thioredoxin reductases 1 (TrxR1) is correlated with the poor prognosis in EGFR wild-type and ALK negative non-small cell lung cancer. Oncotarget.

[39] Dong C, Zhang L, Sun R, Liu J, Yin H, Li X (2016). Role of thioredoxin reductase 1 in dysplastic transformation of human breast epithelial cells triggered by chronic oxidative stress. Sci. Rep..

[40] Zhang W, Zheng X, Wang X (2015). Oxidative stress measured by thioredoxin reductase level as potential biomarker for prostate cancer. Am. J. Cancer Res..

[41] Bai W, Gao J, Qian C, Zhang X (2017). A bioinformatics analysis of differentially expressed genes associated with liver cancer. Chin. J. Hepatol..

[42] Gao H, Hu C, Yu Y, Hu S, Shi C, Wang X (2016). Value of spectral CT-based quantitative analysis in differential diagnosis of liver cancer and liver abscess. Chin. J. Hepatol..

[43] Sivesgaard K, Larsen LP, Sørensen M, Kramer S, Schlander S, Amanavicius N (2018). Diagnostic accuracy of CE-CT, MRI and FDG PET/CT for detecting colorectal cancer liver metastases in patients considered eligible for hepatic resection and/or local ablation. Eur. Radiol..

[44] Sui G-D, Zhang G-Y, Niu Z-J, Hu S-Y (2012). Expression of farnesyltransferase in primary liver cancer. Chin. Med. J.-Peking.

[45] Cong W, Wu M (2015). New insights into molecular diagnostic pathology of primary liver cancer: Advances and challenges. Cancer Lett..

[46] Islami F, Miller KD, Siegel RL, Fedewa SA, Ward EM, Jemal A (2017). Disparities in liver cancer occurrence in the United States by race/ethnicity and state. CA.

[47] Akinyemiju T, Abera S, Ahmed M, Alam N, Alemayohu MA, Allen C (2017). The burden of primary liver cancer and underlying etiologies from 1990 to 2015 at the global, regional, and national level: results from the global burden of disease study 2015. JAMA Oncol..

[48] Simpson RF, Hermon C, Liu B, Green J, Reeves GK, Beral V (2019). Alcohol drinking patterns and liver cirrhosis risk: analysis of the prospective UK Million Women Study. Lancet Public Health.

[49] Ogasawara N, Saitoh S, Denpou H, Kinowaki K, Akuta N, Suzuki F (2020). Poorly differentiated hepatocellular carcinoma in a low-risk patient with an otherwise normal liver. Int. Med..

[50] Kim EK, Song MJ, Jung Y, Lee WS, Jang HH (2019). Proteomic analysis of primary colon cancer and synchronous solitary liver metastasis. Cancer Genom. Proteom..

[51] Engstrand J, Nilsson H, Strömberg C, Mberg C, Jonas E, Freedman J (2018). Colorectal cancer liver metastases—a population-based study on incidence, management and survival. BMC Cancer.

[52] Schirren M, Boeluekbas S, Oguzhan K (2016). Liver and lung metastases of colorectal cancer. Long-term survival and prognostic factors. Chirurg.

[53] Inoue I, Fujii K, Tashiro K (2018). Preoperative chemotherapy may not influence the remnant liver regenerations and outcomes after hepatectomy for colorectal liver metastasis. World J. Surg..

[54] Zhang S, Zhao Y, Zhang M, Wu X (2017). The diagnostic value of tumor markers in bronchoalveolar lavage fluid for the peripheral pulmonary carcinoma. Clin. Respir. J..

[55] Zeng P, Li H, Chen Y, Pei H, Zhang L (2019). Serum CA199 levels are significantly increased in patients suffering from liver, lung, and other diseases. Prog. Mol. Biol. Transl..

[56] Honda K, Katzke VA, Hüsing A, Okaya S, Shoji H, Onidani K (2019). CA19-9 and apolipoprotein-A2 isoforms as detection markers for pancreatic cancer: a prospective evaluation. Int. J. Cancer.

[57] Liu X, Chi X, Gong Q (2015). Association of serum level of growth differentiation factor 15 with liver cirrhosis and hepatocellular carcinoma. PLoS ONE.

[58] Peng W, Zhou Z, Zhong Y, Sun Y, Lu J (2019). Plasma activity of thioredoxin reductase as a novel biomarker in gastric cancer. Sci. Rep..

