# Supplementary material for: Clinical application of thioredoxin reductase as a novel biomarker in liver cancer
Source: Sci Rep. 2021 Mar 16;11:6069. doi: 10.1038/s41598-021-85688-3 (PMC7966739; doi:10.1038/s41598-021-85688-3)
Supplement: Supplementary file 1 — Supplementary Information [file 41598_2021_85688_MOESM1_ESM.docx]

**Clinical application of thioredoxin reductase as a novel biomarker in liver cancer**

Xuping Wu^1^, Qi Wang^2^ ,Yousheng Lu^3^, Jinye Zhang^4^, Hanwei Yin^2^ ,Yongxiang Yi^1^*

Authors Affiliations: ^1^The Second Hospital of Nanjing, Nanjing University of Chinese Medicine, Nanjing, Jiangsu; ^2^Keaise Center for Clinical Laboratory, Wuhan; ^3^Jiangsu Provincial Cancer Hospital & Jiangsu Provincial Cancer Institute & Medical Department of Cancer Hospital Affiliated to Nanjing Medical University, Nanjing; ^4^Nantong Tumor Hospital, Nantong;

*Corresponding Author: Yongxiang Yi, The Second Hospital of Nanjing, Nanjing University of Chinese Medicine, Nanjing 210000, E-mail address: ian0126@126.com

**Running head:** TrxR as a novel biomarker in liver cancer

**Conflicts of a confliction of interest**

Declared no potential conflict of interest for all authors

**Supplemental Experimental Procedures**

**Sample preparation for TrxR activity assessment**

We followed a standard procedure to prepare the human specimens, which was required by the manufacturer’s instruction. Samples from preoperative peripheral blood were collected in EDTA or anticoagulant-free tubes, followed by centrifugation at 3,000 rpm at room temperature for 5 minutes within 2 hours of collection. The supernatant was collected at 4°C and tested immediately. The storage temperature was 4°C, while the incubation temperature was 37°C. The low storage temperature and short handling time have minimized the possibility that the levels of free thiols in the plasma were altered during sample preparation or storage.

**Determination of TrxR activity**

As described in **Materials and methods**, TrxR activity was measured by a commercially available thioredoxin reductase (TrxR) activity colorimetric assay kit (Clairvoyance Health Technology Co., Ltd, Wuhan, China), which is based on DTNB reduction and was carried out according to the manufacturer’s instruction. Positive and negative controls from the kits were included in each reaction to monitor the assay performance.

The calculation of TrxR activity was carried out as follows:

**U/ml = ∆A412/min (thioredoxin reductase) × dil × Vol / (vol × L × ε)**

∆A412/min (thioredoxin reductase) = [∆A412/min (sample) - ∆A412/min (sample + inhibitor)]; dil = sample dilution factor; Vol = volume of reaction in ml; vol = volume of sample in ml; L = length of [optical path](http://www.baidu.com/link?url=qKlH69dtCI0DB-aFzmG_15ePnvC2oXnv-CuTmlytikvnActu4rnwBFBkjBZY2XjoO6dCnqVzRLU1C6xxM0nyXppWEbF8Uzhp5Tww-RU3BN5So2t61EGIn0F6dU6NznMD); ε = extinction coefficient; ∆A412/min (sample)= [A412 (sample, Xmin) - A412/min (sample,0min)] / Xmin; ∆A412/min (sample + inhibitor) = [A412 (sample + inhibitor, Xmin) - A412 (sample + inhibitor,0min)] / Xmin.

The absolute values of absorbances of all patient samples were analyzed by KEA-TR100 analyzer (Clairvoyance, China), and the absolute absorbance linear range at 412 nm was 0.60-3.00. Any absolute absorbance outside this limitation was considered inaccurate and excluded from the study.

**Supplemental Figure S1**


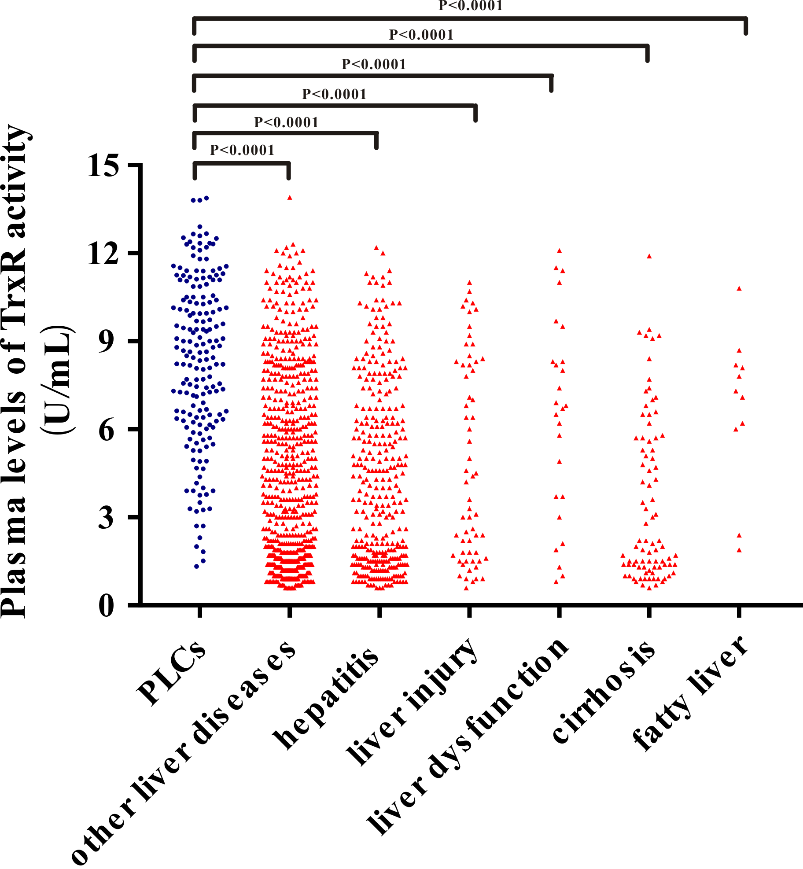


Supplemental Figure S1. Scatter plot of the distribution of plasma TrxR activity levels among primary liver cancer (PLC) and other liver diseases before clinical interventions. P values were calculated by the nonparametric Mann-Whitney U test. Statistical significance was considered as P< 0.05.

**Supplemental Figure S2**


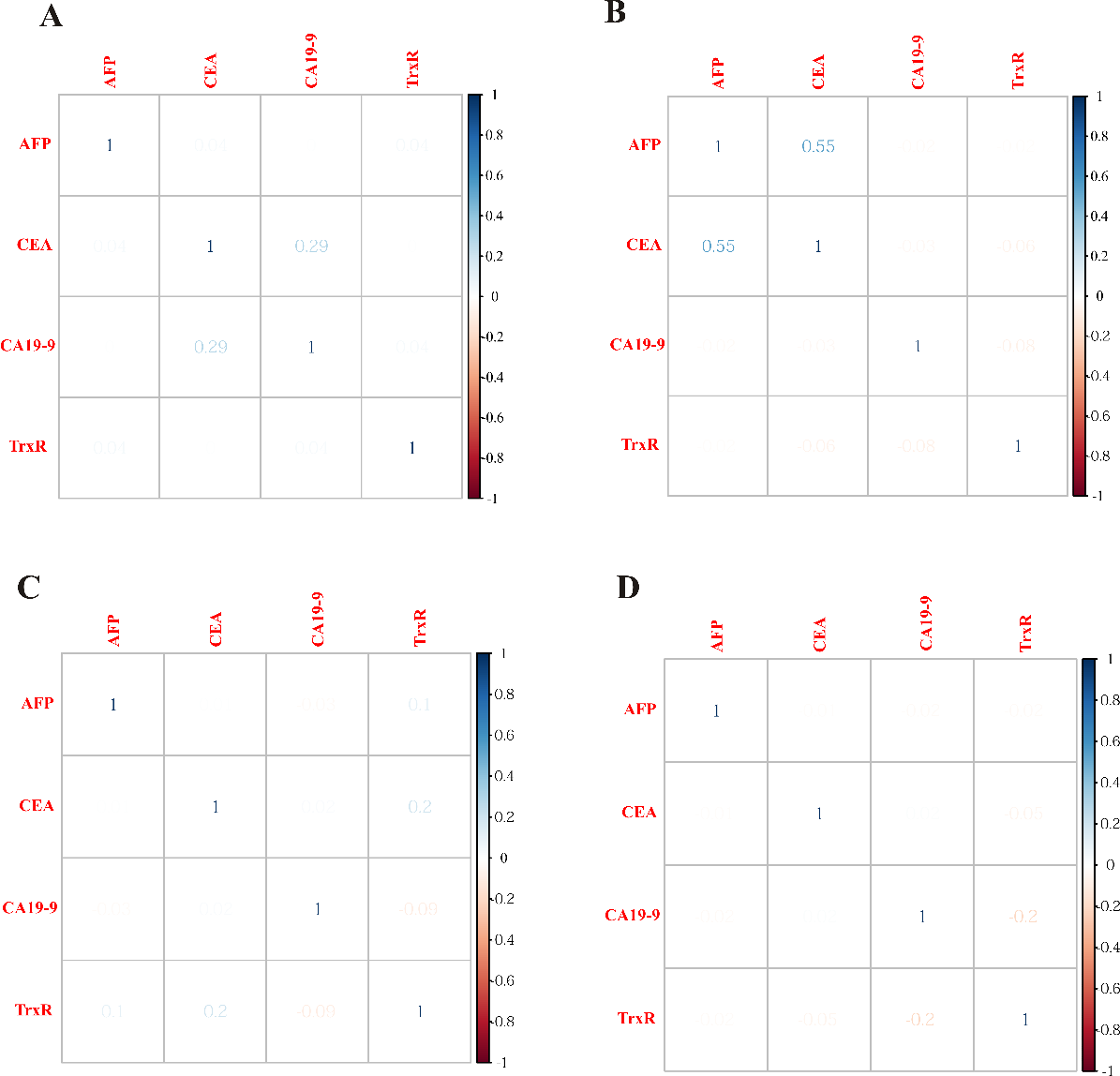


Supplemental Figure S2. (A-D) Correlation analyses of TrxR activity with CEA, CA19-9 and AFP levels in healthy controls (A), liver cancer patients before clinical interventions (B), PLCs after chemotherapy (C), and MLCs after chemotherapy (D). No significant correlation between TrxR activity and CEA, CA19-9, or AFP in either healthy group or liver patients was identified.

**Supplemental Figure S3**


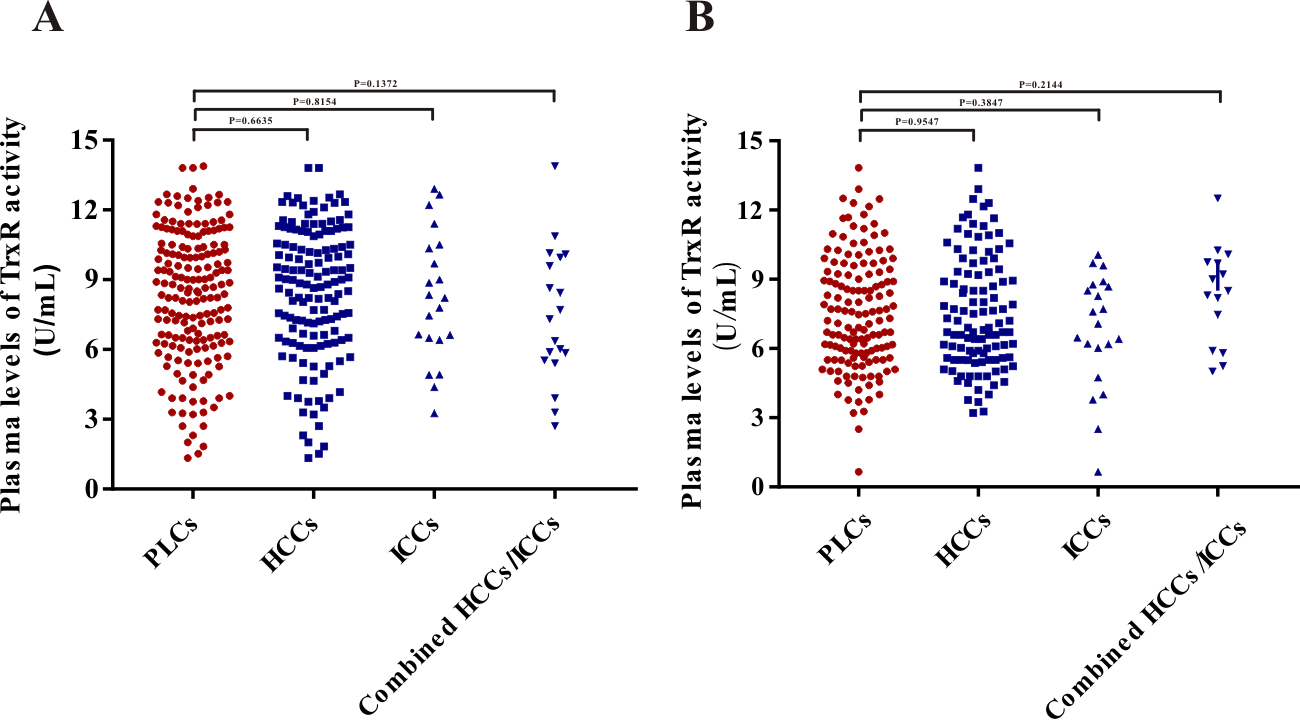


Supplemental Figure S3. (A) Scatter plot of the distribution of plasma TrxR activity levels among different histological types of primary liver cancer (PLC) before clinical interventions, including hepatocellular carcinoma (HCC, n=143), intrahepatic cholangiocarcinoma (ICC, n=21), and combined HCC/ICC (n=19). (B) Scatter plot of the distribution of plasma TrxR activity levels among different histological types of primary liver cancer (PLC) after chemotherapy, including hepatocellular carcinoma (HCC, n=108), intrahepatic cholangiocarcinoma (ICC, n=21), and combined HCC/ICC (n=15). P values were calculated by the nonparametric Mann-Whitney U test. Statistical significance was considered as P< 0.05.

**Supplemental Figure S4**


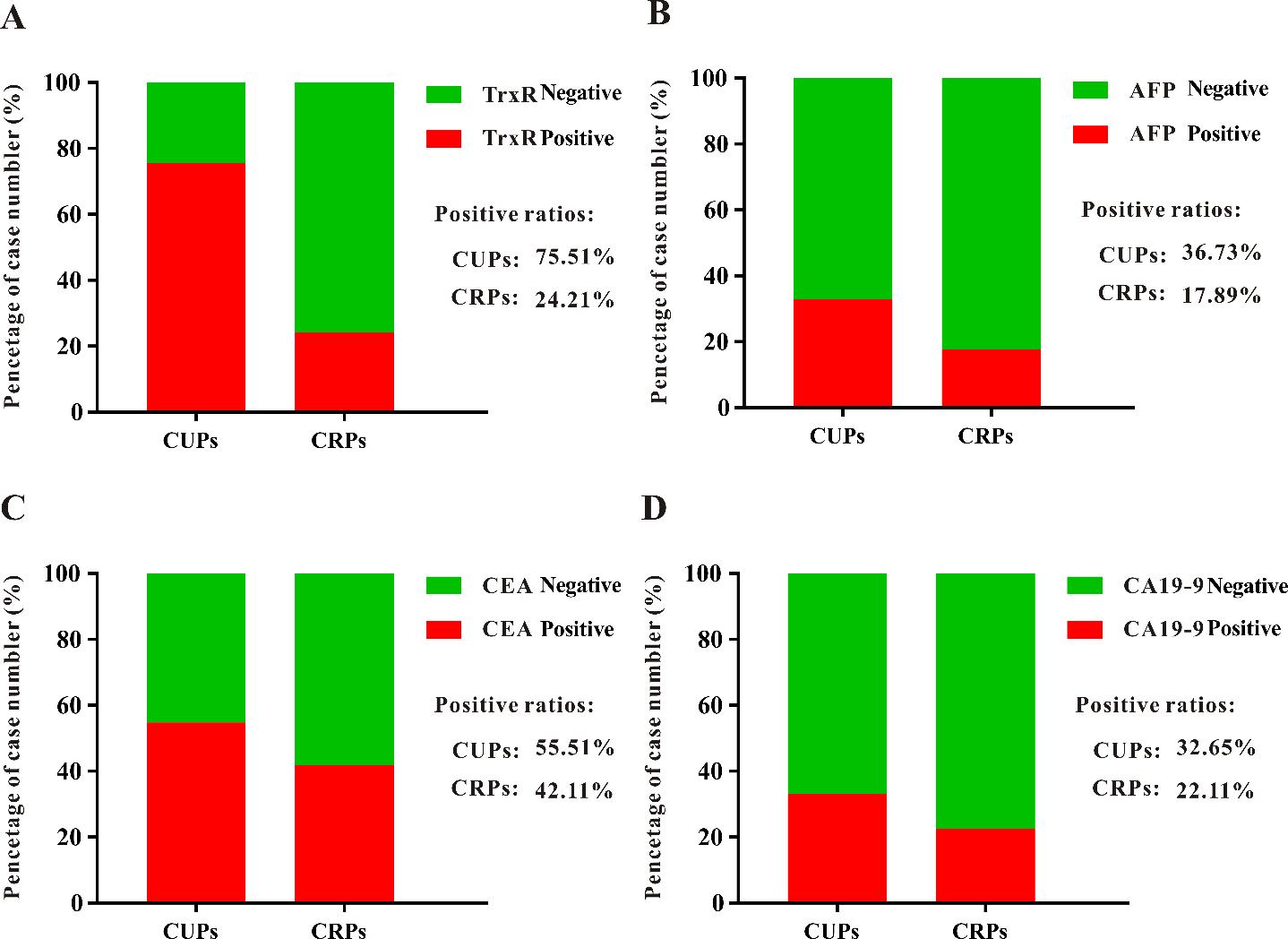


Supplemental Figure S4. (A-D) Sensitivity and specificity of TrxR (SEN:73.47%, SPE:80.00%), AFP (SEN:75.51%, SPE:47.37%), CEA (SEN:36.73%, SPE:87.37%), and CA19-9 (SEN:22.45%, SPE:82.11%) levels based on recommended cut-off values in PLC patients with different clinical outcomes after chemotherapy (CUP vs. CRP). CUP: Clinical Unresponsive Patient; CRP: Clinical Responsive Patient.

**Supplemental Figure S5**


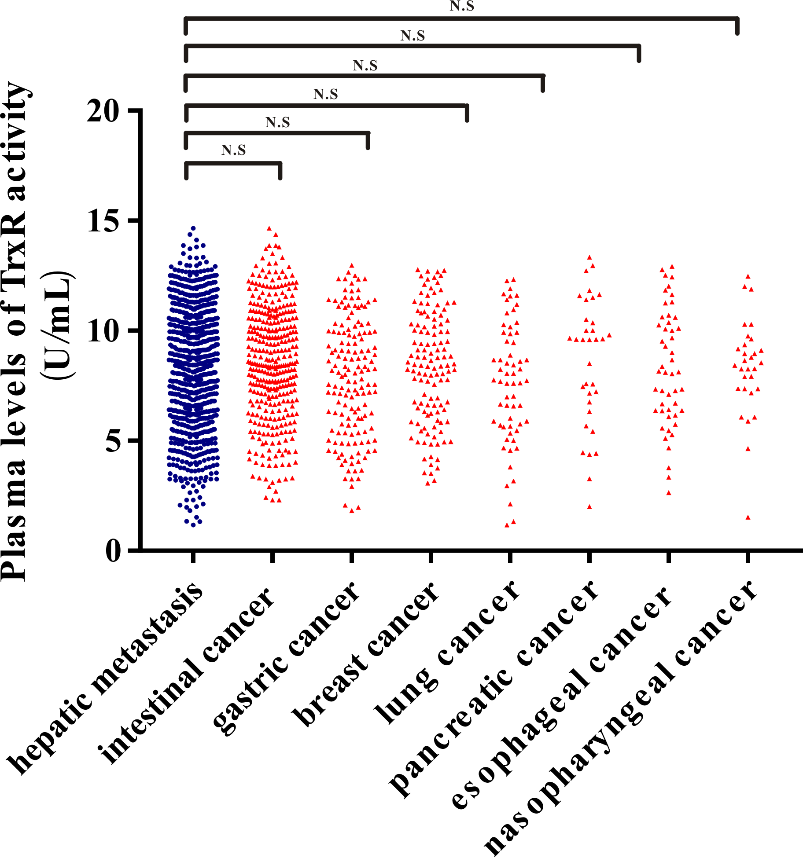


Supplemental Figure S5. Scatter plot of the distribution of plasma TrxR activity levels among MLCs originated from different tumor entities, including intestinal cancer (n=357), gastric cancer (n=154), breast cancer (n=124), lung cancer (n=61), pancreatic cancer (n=34), esophageal cancer (n=49) and nasopharyngeal cancer (n=30). P values were calculated by the nonparametric Mann-Whitney U test. Statistical significance was considered as P< 0.05. N.S: no statistical significance.

**Supplemental Figure S6**


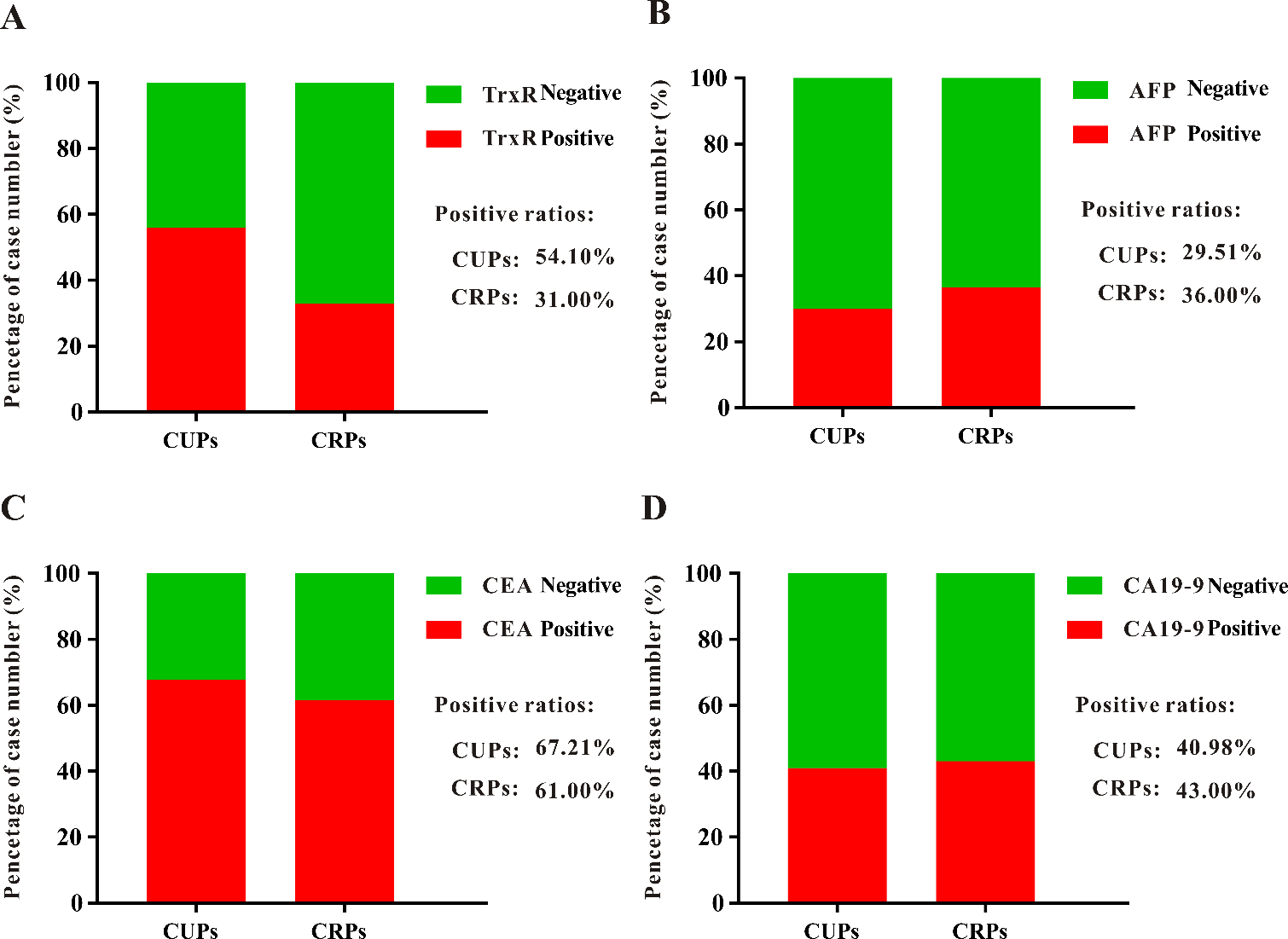


Supplemental Figure S6. (A-D) Sensitivity and specificity of TrxR (SEN:63.93%, SPE:63.00%), AFP (SEN:57.38%, SPE:58%), CEA (SEN:13.11%, SPE:97.00%), and CA19-9 (SEN:24.59%, SPE:84.00%) levels based on recommended cut-off values in MLC patients with different clinical outcomes after chemotherapy (CUP vs. CRP). CUP: Clinical Unresponsive Patient; CRP: Clinical Responsive Patient.

**Supplemental Table S1**

| **Characteristics** | **Before clinical intervention** | **PLCs** | **MLCs** | **Healthy controls** |
| --- | --- | --- | --- | --- |
|  |  | **After chemotherapy** | |  |
| N | 183 | 144 | 809 | 150 |
| Age（IQR,years） | 63 (55-69) | 61 (52-67) | 61 (51-68) | 67 (63-76) |
| **Gender**（**%**） | | | | |
| Male | 106 (57.9) | 114 (79.2) | 372 (46.0) | 76 (55.4) |
| Female | 77 (42.1) | 30 (20.8) | 437 (54.0) | 74 (44.6) |
| **Histologiacal type**（**%**） | | | | |
| Hepatocellular carcinoma（HCC） | 143 (78.1) | 108 (75.0) | — | — |
| Intrahepatic cholangiocarcinoma （ICC） | 21 (11.5) | 21 (14.6) | — | — |
| Combined HCC/ICC | 19 (10.4） | 15 (10.4) | — | — |
| **TNM**（**%**） | | | | |
| Ⅰ-Ⅱ | 129 (70.5) | 57 (39.6) | 0 (0.0) | — |
| Ⅲ | 54 (29.5) | 76 (52.8) | 10 (1.24) | — |
| Ⅳ | 0 (0.0) | 11 (7.6) | 799 (98.76) | — |
| **Distant metastasis**（**%**） | | | | |
| No | 183 (100.0) | 133 (92.4) | 10 (1.24) | — |
| Yes | 0 (0.0) | 11 (7.6) | 799 (98.76) | — |

Supplemental Table S1. The characteristics of liver cancer cases and healthy controls in the study.
